# A Plant Leaf Geometric Parameter Measurement System Based on the Android Platform

**DOI:** 10.3390/s19081872

**Published:** 2019-04-19

**Authors:** Haiyun Liu, Xu Ma, Ming Tao, Ruoling Deng, Kemoh Bangura, Xiangwu Deng, Chuang Liu, Long Qi

**Affiliations:** 1 College of Engineering, South China Agricultural University, Guangzhou 510642, China; haiyun0420@stu.scau.edu.cn (H.L.); maxu1959@scau.edu.cn (X.M.); taoming@stu.scau.edu.cn (M.T.); drl1207@stu.scau.edu.cn (R.D.); bangura.kemoh@yahoo.com (K.B.); dengxiangwu123456@163.com (X.D.); liuchuang@stu.scau.edu.cn (C.L.); 2 Institute of Intelligent Machines, Chinese Academy, Hefei 230031, China

**Keywords:** plant leaf, image analysis, Android system, dynamic reference

## Abstract

Automatic and efficient plant leaf geometry parameter measurement offers useful information for plant management. The objective of this study was to develop an efficient and effective leaf geometry parameter measurement system based on the Android phone platform. The Android mobile phone was used to process and measure geometric parameters of the leaf, such as length, width, perimeter, and area. First, initial leaf images were pre-processed by some image algorithms, then distortion calibration was proposed to eliminate image distortion. Next, a method for calculating leaf parameters by using the positive circumscribed rectangle of the leaf as a reference object was proposed to improve the measurement accuracy. The results demonstrated that the test distances from 235 to 260 mm and angles from 0 to 45 degrees had little influence on the leafs’ geometric parameters. Both lab and outdoor measurements of leaf parameters showed that the developed method and the standard method were highly correlated. In addition, for the same leaf, the results of different mobile phone measurements were not significantly different. The leaf geometry parameter measurement system based on the Android phone platform used for this study could produce high accuracy measurements for leaf geometry parameters.

## 1. Introduction

Leaves are the main plant organs that perform photosynthesis, transpiration, and the synthesis of organic matter [1]. Leaf geometric parameters are not only an important indicator of plant growth and development, yield formation, and a variety of other characteristics, but also provide important data support for the cultivation and management of crops, and the monitoring of pests and diseases [2]. Therefore, accurately measuring geometric parameters such as length, width, perimeter, and area of the leaf has significant agronomic implications.

In the past, studies have been focused on determining the leaf area of plants as opposed to leaf length, width, and perimeter. Some of the traditional methods used to measure the geometric parameters of plant leaves include direct measurements, grid counting methods [3], graph decomposition, and coefficient methods [4]. However, the desktop leaf area meter is not always available due to its expensive cost and high maintenance requirements, whereas the hand-held leaf area meter is complicated to operate with low precision [5]. In contrast, the image analysis method has the features of relatively simple operation, high accuracy, and low cost. In recent years, with Android smartphone popularity due to its portability and easy operation, many researchers have used the Android system’s mobile platform for agroforestry measurements. Qu et al. [6] designed an expert diagnosis system based on Android phones, which could diagnose diseases and pests. The system obtained images through the mobile phone’s camera; the server reasoned mechanisms to diagnose pests; and the results were fed back to the mobile phone users. Gong et al. [7] developed an application for android smartphones that could estimate citrus yields. They used a phone to obtain an image of a tree and used image processing techniques for identifying the fruits in the image. Zhong et al. [8] proposed an application based on the Android platform for automatic plant identification, which was able to identify 126 tree species. The application could capture high-quality photographs of leaves, and was very useful for users to identify tree species. 

A new method for measuring the geometric parameters of plant leaves based on the Android mobile phone was also developed. Gong et al. [9] developed a non-destructive measurement software for plant leaf areas. The software was based on the Android platform and used Java language, combined with an image processing library. However, the software required drawing a closed ring around the leaf with a touch pen after the leaf was photographed, which was troublesome to operate. Guo et al. [10] developed a non-destructive measurement system of plant leaf areas based on the Android mobile phone platform. A geometric correction method was proposed, and the functions of image acquisition, self-selected image, and color contrast were designed. The experimental results showed that the system was not limited by the leaves’ shape, but the accuracy was not high.

Current leaf geometric parameter measurements are limited to only leaf area measurements. Also, most existing image processing methods require users to understand the relevant software operations. Furthermore, image acquisition generally requires the use of a special device, and the device needs to maintain a fixed distance from the leaf and parallel to the leaf, which is difficult to operate. With the popularity of smartphones, a leaf geometry measurement software based on smart phones has been developed, but no portable measuring systems have been developed, and the current measurement accuracy is low. The primary objective of this study was to propose the Android mobile phone system for accurate and convenient leaf area and other leaf geometric parameters measurements, which minimize the effects of distance, angle, leaf shape, and environment, overcoming the disadvantages of previous methods and instruments. The secondary objective was to compare the performance of the Android mobile phone with other types of mobile phones.

## 2. Materials and Methods

### 2.1. Measuring Device

Camera type and resolution of different mobile phones are different. These differences may have a certain impact on the measurement results. Therefore, four types of mobile phones were used in the test to investigate the adaptability of the developed Android system. The specifications of the phones are shown in Table 1.

**Figure 1:**
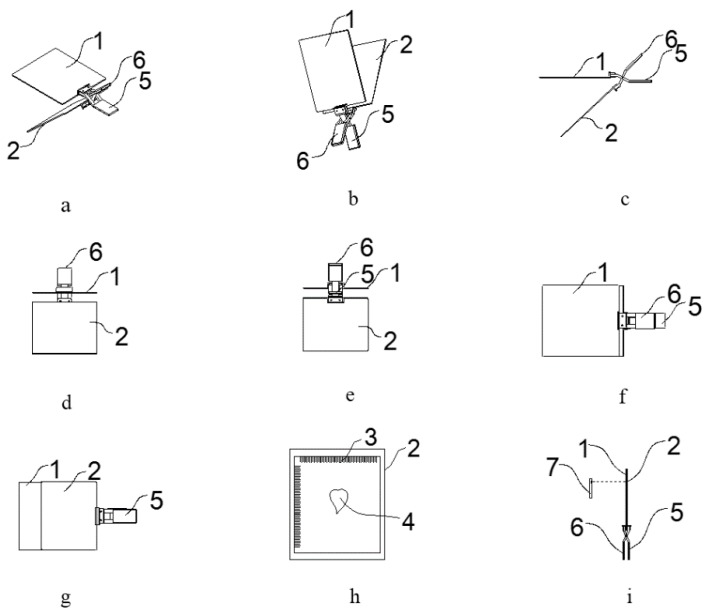
The leaf measuring device. (**a**) Perspective structural view of one of the angles; (**b**) perspective structural view of another angle; (**c**) front view; (**d**) left side view; (**e**) right side view; (**f**) plane view; (**g**) bottom view; (**h**) structural view of the leaf fixing device in which the correction plate is fixed to the lower splint; and (**i**) schematic diagram of the image acquisition system. (**1**) upper splint; (**2**) lower splint; (**3**) correction plate; (**4**) measured leaf; (**5**) first handle; (**6**) second handle; and (**7**) mobile terminal.

The leaf measuring device used in the test is illustrated in Figure 1. The device was composed of three main components: upper splint, lower splint, and correction plate. The upper splint was a transparent acrylic plate and the lower splint was a pure black plate. The upper splint could be opened or closed relative to the lower splint. The upper and lower splints constituted a clamp. The device also included a first and second handle. The first handle was fixed on the upper splint, the second handle was fixed on the lower splint, and the first handle was hinged with the second handle. The first surface of the calibration plate had a black rectangular frame with four sides parallel to the four sides of the correction plate, and a standard millimeter tick mark was engraved on the upper boundary and the left boundary of the black rectangular frame. The millimeter tick mark was used to calculate the positive circumscribed rectangular parameters of the leaf during measurement. The second surface of the correction plate was fixed on the upper surface of the lower splint. The upper splint and the lower splint were clamped and fixed by holding the first handle and the second handle. When working, the leaf was flattened and placed in the rectangular frame on the calibration plate. There was no coincidence with the millimeter scale line. The two handles were closed together, so that the measured leaf was fixed and kept flat.

### 2.2. Experimental Methods

To verify the repeatability, accuracy, and applicability of leaf geometric parameters tested by the software discussed later in the paper, the following experiments were carried out. In the following experiments, the elongation axis of all measured objects was as parallel as possible to the either sides of the correction plate.

#### 2.2.1. Measurement of Rule Graphics

**Figure 2:**
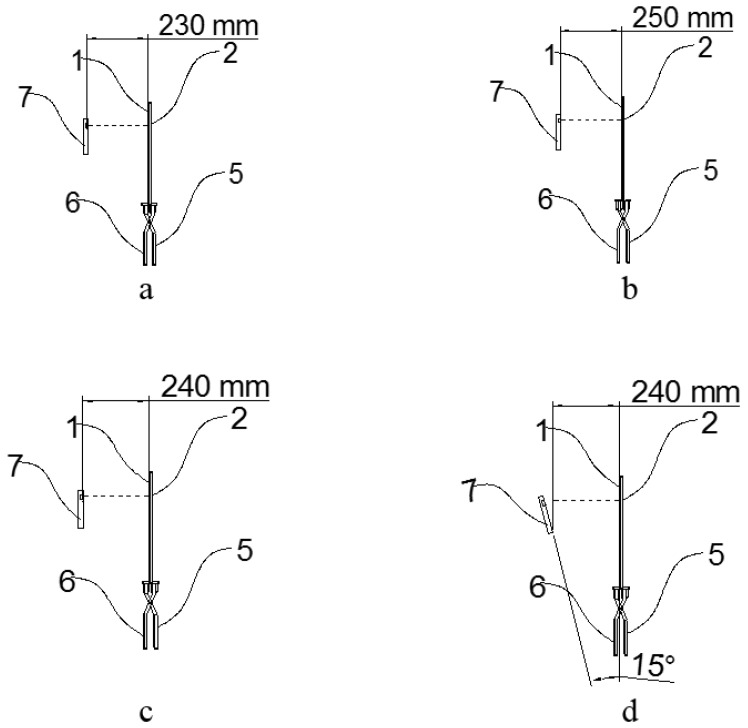
Distance and angle test chart. (**a**) distance from the mobile phone plane to the measuring device plane (230 mm); (**b**) distance from the mobile phone plane to the measuring device plane (250 mm); (**c**) angel between the mobile phone plane and measuring device plane (0 degree); and (**d**) angel between the mobile phone plane and measuring device plane (15 degrees).

Regular graphics were accurately drawn and cut into different shapes, namely square, rectangle, circle, and triangle to represent different leaf shapes. The specific shapes and sizes of the regular leaf graphics are presented in Table 2. To study the influence of different test distances on measured results, seven distances from the mobile phone plane to the measuring device plane were selected, ranging from 230 to 260 mm, at 5-mm intervals. The HUAWEI nova 1 mobile phone listed in Table 1 was used to measure graphical geometry parameters. Figure 2a,b shows the distances from the mobile phone plane to the measuring device plane.

**Figure 3:**
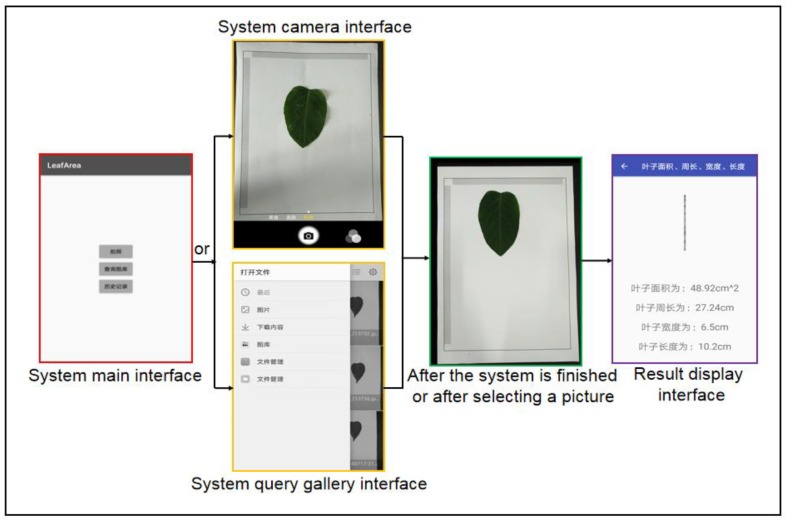
Running process of the software chart.

Figure 3 demonstrates a typical process of a test. Each rule graphic was measured ten times at the same test distance, and then the average value of the ten readings was obtained. The error between the true and measured values was calculated. To study the effect of the deflection angle changes on the results, the angle between the camera plane and the plane of the measuring device was changed to 0, 15, 30, and 45 degrees under a constant test distance of 240 mm. Relative errors of the leaf geometric parameters at the test distance and angle were recorded. Figure 2c,d show the angels between the mobile phone plane and measuring device plane.

#### 2.2.2. Measurement of the Leaf

Cucumber, soybean, tomato, and rice leaves were selected for measurements. These four different crop leaves represented four different plant leaf shapes. Cucumber leaves represented heart shape, soybean leaves represented oval shape, tomato leaves represented sawtooth edge shape, and rice leaves represented slender shape. All the leaves were collected from the College of Resource and Environmental Engineering, South China Agricultural University. The test was carried out in lab. In addition to the measurement of geometric parameters, comparative experimental measurements were also carried out, in which the length and width of the leaves were directly measured using a Vernier caliper. The area was measured by the grid method. Ten leaves of different sizes were selected for each plant. The HUAWEI nova 1 mobile phone was used to measure leaf geometry parameters. The measurement was repeated ten times for each leaf and the measured value was taken. The results were used to test the measurement accuracy of the system. For outdoor measurements, 20 leaves were randomly selected. The test site was at College of Engineering, South China Agricultural University. The weather was cloudy with occasional sun. The HUAWEI nova 1 mobile phone was used to measure leaf geometry parameters. The details of the test were similar to the lab measurements.

#### 2.2.3. Different Mobile Tests

Twelve leaves were randomly selected from the College of Engineering, South China Agricultural University. The tests were carried out in the lab. The four types of mobile phones described in Table 1 were used in the tests to measure leaf geometry parameters. The measurement was repeated ten times for each leaf using each of the four mobile phones, and then used the standard method to calculate the actual geometric parameters of the leaves. The results were used to test the applicability of the system.

### 2.3. Implementation of the Software

**Figure 4:**
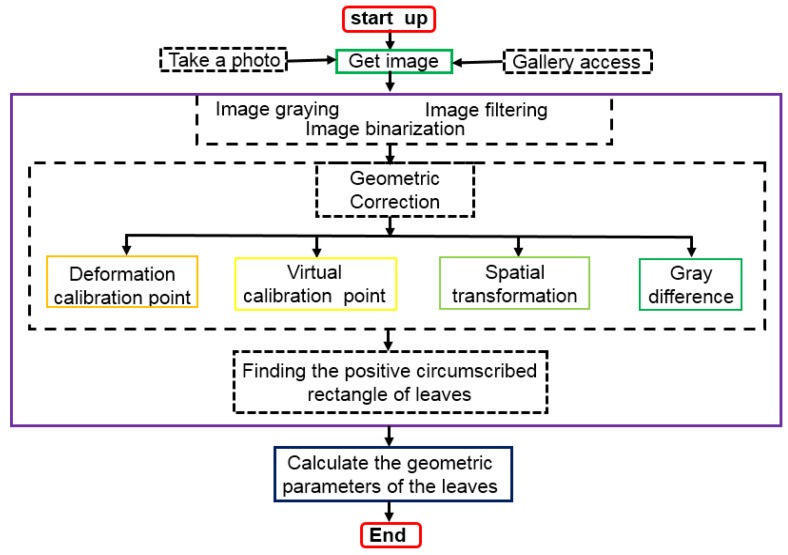
Software development process chart.

Figure 4 shows a detailed software development process. Details of the design of the software are discussed in the following sections.

#### 2.3.1. System Software Platform

Under the Windows 7 operating system, an application development environment based on the Android SDK (Java development kit) + Java JDK 8 (Java development kit) + Android studio 3.0 + ADT (Android development tools) was built, and the system software was developed for Android 7.0 and above [11].

#### 2.3.2. Image acquisition and processing

The software was able to call the camera to capture images, and obtain the gallery image by obtaining the file directory of the memory card. After acquiring the image, the system automatically implemented the image processing. The specific steps were as follows: changing the color image into a grayscale image by the weighted average method [12,13,14], smoothing the resultant grayscale image by the Gaussian kernel filter (1 × 1 pixels zero mean), and converting the images into binary image [15,16,17].

#### 2.3.3. Image Geometry Calibration

**Figure 5:**
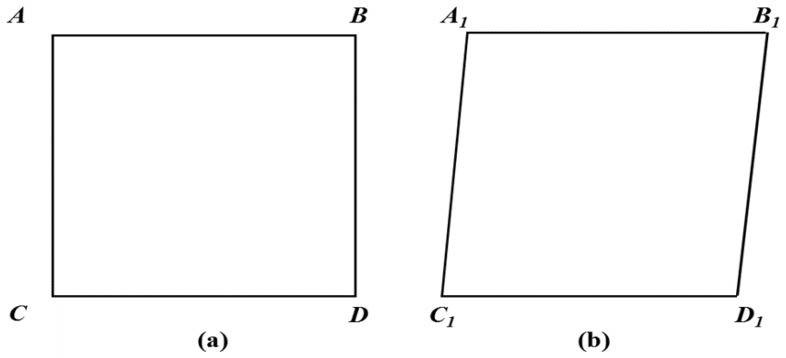
(**a**) Calibration pattern and (**b**) distortion pattern.

To better reflect the real image, geometric distortion calibration was performed. The Hough transformation method was used to find the four distortion calibration points of the rectangular calibration plate in the distorted image [18]. The system first found the outline of the rectangular calibration plate, and then used the approxPolyDP function to specify the precision to approximate the contour of the rectangular calibration plate, and fit the points on the contour of the rectangular calibration plate to four corner points. The four corner points were the distortion calibration points of the distorted image, and the coordinates of the four deformation calibration points were obtained (Figure 5).

At the same time, a virtual rectangular calibration plate was established in the Java program and determined a virtual calibration point corresponding to the calibration point and the distortion calibration point on the virtual rectangle calibration plate. The virtual rectangular calibration plate was the virtual orthogonal projection of the rectangular calibration plate without deformation, in which the coordinates of each virtual calibration point were determined according to the coordinates of the calibration point of the rectangular calibration plate before the geometric deformation occurred.

By matching the coordinates of each deformation calibration point with those of the corresponding virtual calibration point, the image transformation matrix was obtained as follows:

(1) $$\left[\begin{array}{c} u\\ v\\ 1 \end{array}\right]=\lambda \left[\begin{array}{ccc} A & B & C\\ D & E & F\\ G & H & 1 \end{array}\right]\left[\begin{array}{c} x\\ y\\ 1 \end{array}\right]\Rightarrow \{\begin{array}{c} u=Ax+By+C\\ v=Dx+Ey+F\\ 1=Gx+Hy+1 \end{array}$$

where (*x*, *y*) is the coordinate of the distortion calibration point on the distorted image, (*u, v*) is the coordinate of the virtual calibration point on the virtual calibration plate, *A–H* is the parameter in the image transformation matrix, and *λ* is the image scale, and the value is 1. By solving the value of the parameters, *A–H*, and calculating a product of the distortion image and the inverse matrix of the image transformation matrix, a calibrated image was obtained.

### 2.4. Analyses of Leaf Parameters

In this study, the positive circumscribed rectangle of the leaf was proposed as the reference object of the leaf, which avoided measurement errors caused by other larger or smaller reference objects, and improved the measurement accuracy.

#### 2.4.1. Positive Circumscribed Rectangle of Leaf

After grayscale transformation, filtering, binarization, the maximum and sub-large contour operations were carried out on the calibrated images. Only the contours of the two connected regions of the leaf and the black rectangular frame were left in the image, and the contours were traversed from left to right. The leftmost and rightmost pixels of each contour in the image were obtained and their corresponding abscissas *X_min_* and *X_max_* were recorded. The contours from bottom to top were traversed to obtain the bottommost and topmost pixels in the image [19,20,21], and the ordinates *Y_min_* and *Y_max_* were recorded. The length and width of the positive circumscribed rectangle were obtained from Equations (2) and (3), respectively:

(2) $$Height=Y_{\max}-Y_{\min}$$

(3) $$Length=X_{\max}-X_{\min}$$

**Figure 6:**
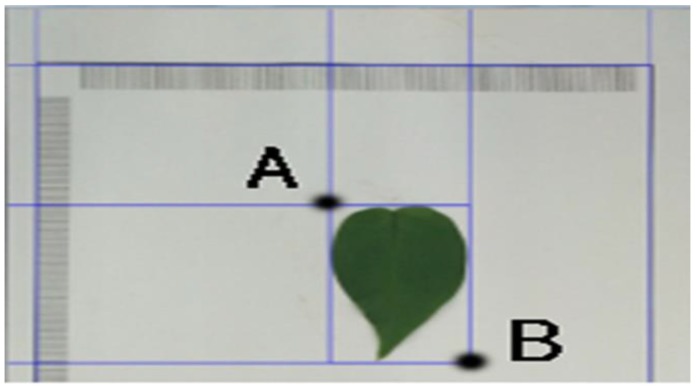
Positive circumscribed rectangle composed of A and B.

A point *A* was used to represent the coordinates (*X_min_*, *Y_min_*), and *B* represented the coordinates (*X_max_*, *Y_max_*). Then, *A* and *B* formed a positive circumscribed rectangle, which was used to identify the measured leaf or the black rectangular frame as shown in Figure 6.

#### 2.4.2. Calculation of Leaf Length and Width

After obtaining the coordinates of the four corner points of the positive circumscribed rectangle of the leaf, the next step was to draw the extension line of each side of the positive circumscribed rectangle according to the coordinates of the two corner points, and the final step was to find the intersection coordinates of each extension line and the black rectangle. The number of tick marks between the two intersection points was calculated, obtaining the length and width of leaf. As the tick marks are thin, some tick marks may be broken after binarization, which may lead to an increase in measurement error. Therefore, the morphological processing (dilate) was used to solve this problem [22]. The extension lines on the left and right sides of the positive circumscribed rectangle intersected the black rectangular frame at two points, the tick marks between the two intersection points were parallel to the *Y* axis, and the interval between the tick marks was small. Traditional expansion is easy to dilate two scale lines into one. In this study, *Y*-axis expansion was adopted, and the core size was (1, 49), and the effect was good. On the tick marks parallel to *X*-axis, *X*-axis was used, and the core size was (49, 1). If the extension line of the circumscribed rectangle was between the two tick marks, the number of tick marks between the two intersection points was taken as the length or width of the leaf, and the length between the extension line and the tick mark was ignored, which resulted in measurement error of the leaf geometric parameters. The length between the extension line and the tick mark was calculated by multiplying the number of pixels between the extension line and the tick mark and the length per pixel. The actual size of the leaf length and width could be obtained by subtracting one of the number of tick marks and adding the length between the extension line and the tick mark.

#### 2.4.3. Calculation of Leaf Area and Perimeter

This study used the contour of the outermost layer of the extracted leaf to calculate the contour area to eliminate the influence of lesions and wormholes on the leaf area calculation [23,24]. The leaf area was obtained according to the proportional relationships between the total number of pixels in the leaf contour, the total number of pixels in the positive circumscribed rectangle of the leaf, and the area of the positive circumscribed rectangle of the leaf [25]. The leaf perimeter was obtained from the proportional relationship between the total number of pixels on the leaf contour, the total number of pixels on the positive circumscribed rectangle of the leaf, and the circumference of the positive circumscribed rectangle of the leaf [26,27,28]. Calculations were done using the following formulas:

(4) $$S=S_{r}\times \frac{P_{t}}{P_{rt}}$$

(5) $$S_{r}=L_{r}\times W_{r}$$

(6) $$C=C_{r}\times \frac{P_{c}}{P_{ct}}$$

(7) $$C_{r}=(L_{r}+W_{r})\times 2$$

where *S* is leaf area; *S_r_* is the area of the positive circumscribed rectangle of the leaf; *Pt* is the total number of pixels within the leaf contour; *P_rt_* is the total number of pixels in the contour of the positive circumscribed rectangle of the leaf; *L_r_* is the length of the positive circumscribed rectangle of the leaf; *W_r_* is the width of the positive circumscribed rectangle of the leaf; *C* is leaf perimeter; *C_r_* is the perimeter of the positive circumscribed rectangle of the leaf; *P_c_* is the total number of pixels on the leaf contour; and *P_ct_* is the total number of pixels on the contour of the positive circumscribed rectangle of the leaf.

## 3. Results

### 3.1. Distance Tests of Rule Graphics

**Figure 7:**
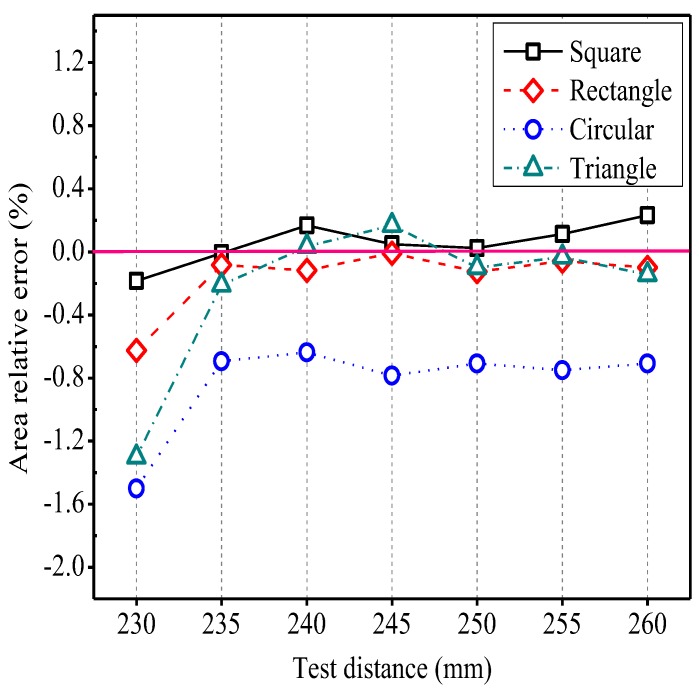
The relationship between the test distance and area relative error for different leaf shape patterns.

Different test distances significantly influenced the area measurements of regular graphics, the results are showed in Figure 7. Regardless of the shape of the graphic, it was found that the relative error was maximum when the test distance was set at 230 mm, and decreased when the test distance was increased to 235 mm. With the continuous increase in test distance, the measurement error tended to flatten out completely. It showed that the relative error of square area ranged from −0.184% to 0.232%, the relative error of rectangular area ranged from −0.625% to −0.008%, the relative error of circular area ranged from −1.500% to −0.637%, and the relative error of triangle area ranged from −1.300% to 0.167%. At the same time, different shapes of the graphics also affected the accuracy of measured areas. It can be seen that the relative error of square area and the relative error of rectangular area were smaller than those of circular and triangle areas, and the circular graphic produced the worst results. Therefore, the results were influenced by graphic shape, and the more the shape approached a rectangle, the better the accuracy of measured area was given.

The relationship between the changes in test distance and measured perimeter, length, and width showed similar effects to those of Figure 7. After measuring the other geometric parameters of each shape, the trend of its relative error was found to be the same as the relative error of area, and the relative errors were relatively small (results are not shown).

### 3.2. Angle Tests of Regular Images

**Figure 8:**
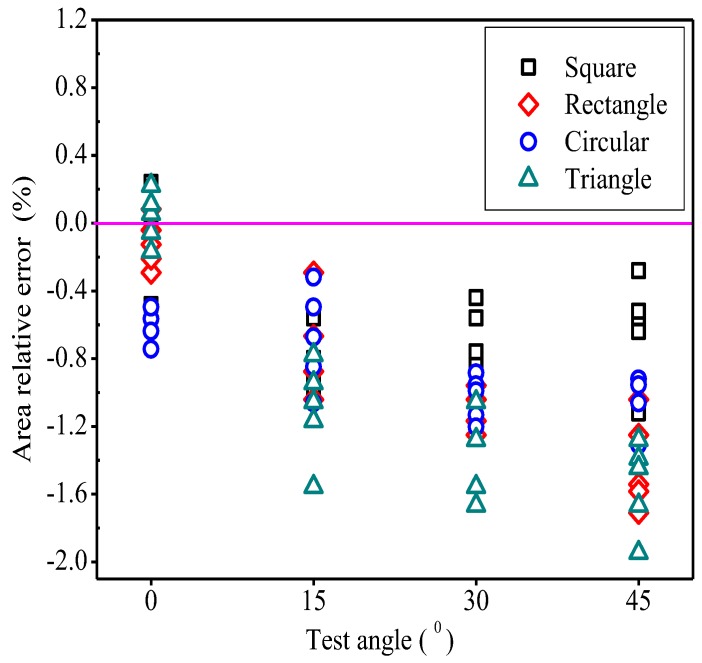
The relationship between the test angle and the area error of different shape patterns.

Different test angles influenced area measurements of regular graphics. As the test angle increased, the relative error of the measured area increased, and the range of the relative error of measured area increased as well (Figure 8). In terms of numerical value, the change in test angle had little impact on the relative error of area. At 0°, the relative error ranged from −0.48% to 0.24% for the square area, −0.292–0.083% for the rectangular area, −0.743 to −0.495% for the circular area, and −0.167–0.222% for the triangular area. At 45°, the corresponding relative errors were −1.120 to −0.640%, −1.708 to −1.042%, −1.309 to −0.920%, and −1.944 to −1.278%. It can be seen that the relative errors were either positive or negative. If the plane of the mobile phone’s camera was not parallel to the plane of the blade, the relative errors were negative, meaning that the measured values by software were smaller than the actual values.

The relationships between the variation of test angle and the measured perimeter, length, and width were similar to those of Figure 8, but the amplitudes of variation and the ranges of value were smaller. The results further explained that image distortion had little effects on the measurement accuracy of this system.

### 3.3. Lab Leaf Tests

**Figure 9:**
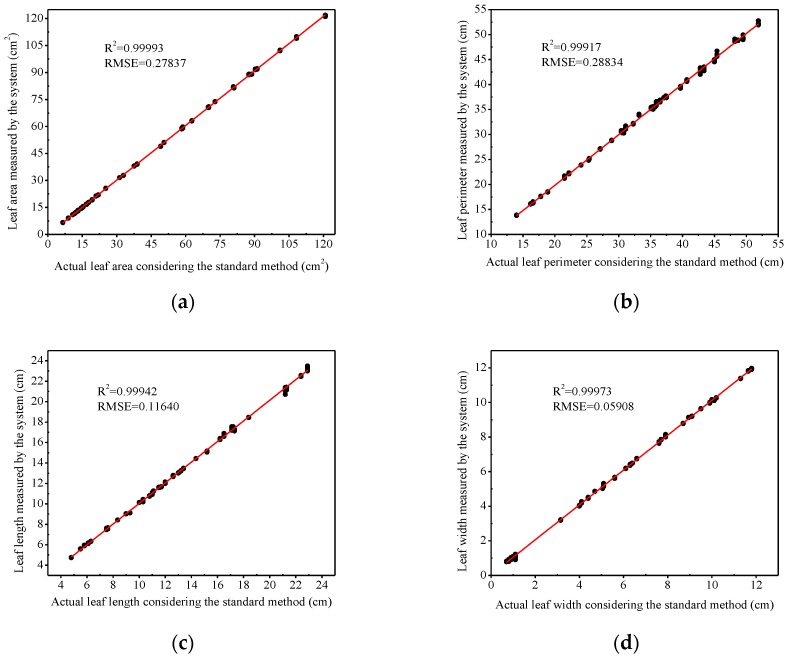
The relationship between leaf geometry parameters measured by the system and the grid method. (**a**) Leaf areas; (**b**) leaf perimeters; (**c**) leaf lengths; and (**d**) leaf widths.

The leaf area values of cucumber, soybean, tomato, and rice measured were compared with those analyzed by the developed system, and the results are presented in Figure 9a. The coefficient of determination (*R*^2^) was quite high (0.99993), and the root mean square error (RMSE) was 0.28. In addition, the results of the perimeter, length, and width values of the plant leaves were compared with those measured using the Vernier caliper line method. The comparisons showed that the values *R*^2^ for the perimeter, length, and width of the different plant leaves were all higher than 0.99. The corresponding RMSE values were 0.29, 0.12, and 0.06.

**Figure 10:**
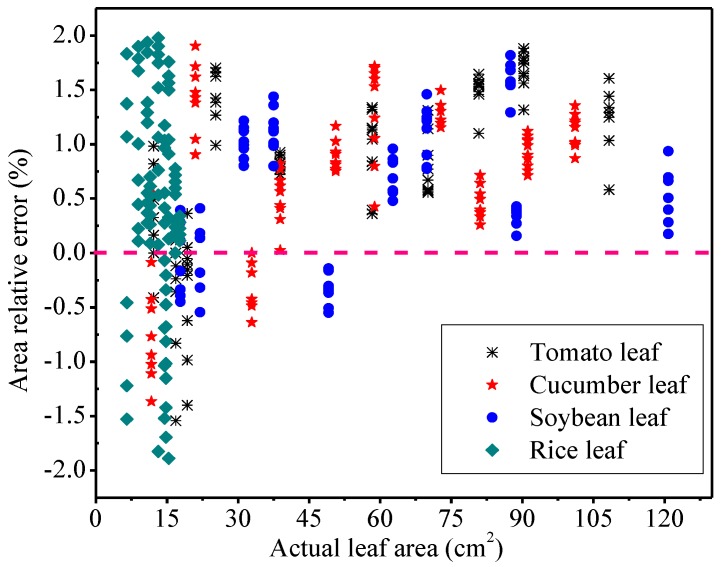
The relationship between the relative errors of the leaf areas measured by the system and the grid method.

Furthermore, the relative error range of the different leaf areas measured by the developed system and that of by the grid method were plotted together (Figure 10). It was noted that the influence of the leaf shapes on the measured area error was not large. The ranges of the relative error of leaf area were −1.37–1.72% for cucumber leaves, −0.62–1.70% for soybean leaves, −1.60–1.80% for tomato leaves, and −2.00–2.00% for rice leaves. Most of the relative errors were positive values, which means that the area value measured by the developed system was mostly larger than the actual value of the leaf area. Moreover, the measured value was larger than the actual value when the leaf area was larger than 50 cm^2^. The relative error of rice leaf area was relatively larger than other leaves. These results indicated that the performance of the developed system to measure slender leaves was inferior to broad leaves.

### 3.4. Outdoor Leaf Tests

**Figure 11:**
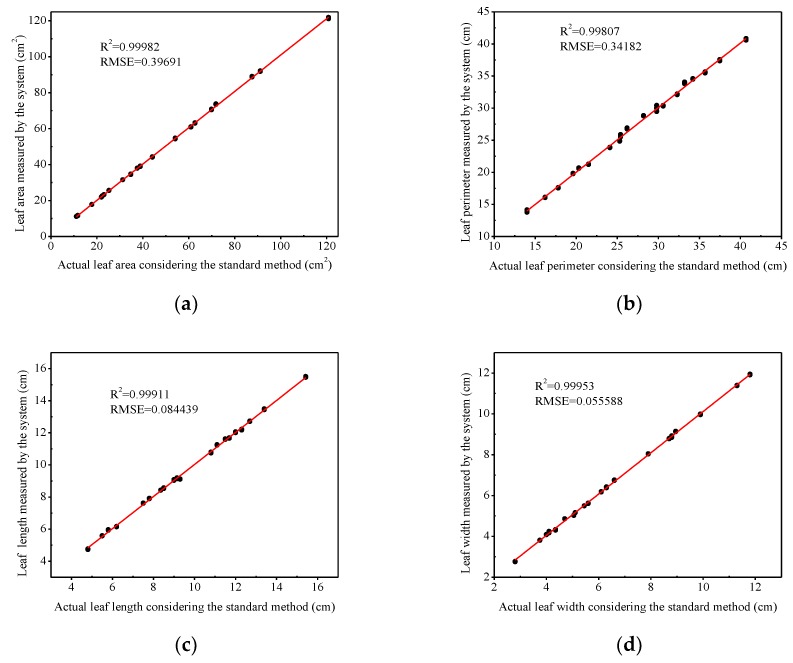
The relationship between leaf geometric parameters measured by the traditional method and by the developed system. (**a**) Leaf areas; (**b**) leaf perimeters; (**c**) leaf lengths; and (**d**) leaf widths.

The area values of all leaves measured by the grid method were compared with those measured by the developed system (Figure 11a). The coefficient of determination *R*^2^ value (0.99982) by the two methods was also very high, and its RMSE was 0.40. In addition, the perimeter, length, and width values of the plant leaves measured by the Vernier caliper line method which was considered to be the most accurate, were compared with values measured by the developed system, and the results were presented in Figure 11b–d, respectively. The coefficient of determination *R*^2^ of the perimeter, length, and width values of the different plant leaves by the two methods were 0.99807, 0.99911, and 0.99953, respectively, and the corresponding RMSE values were 0.34, 0.08, and 0.06.

**Figure 12:**
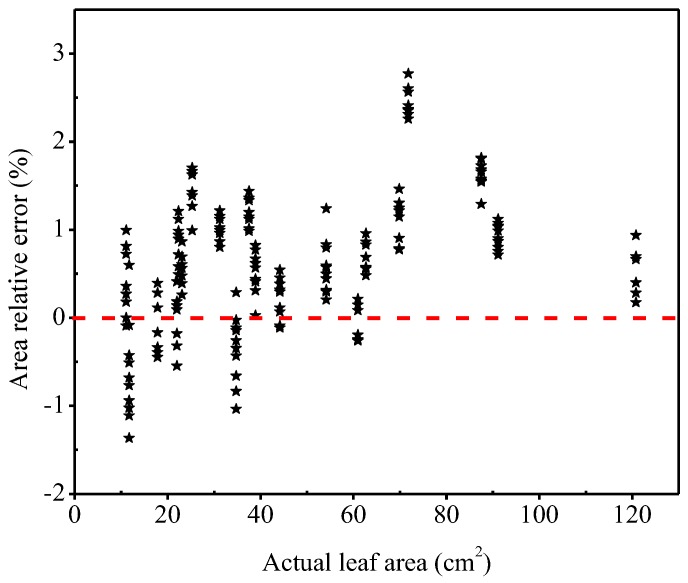
The relationship between the relative error of the leaf area measured by the developed system and the leaf area measured by the grid method.

The relative error range of the different leaf area measured by the developed system and that of by the grid method were also compared (Figure 12). The influence of leaf size on the relative error of measured leaf area was not significant. The relative error of the measured area for all leaves ranged from −1.37% to 2.52%. Most of area values measured by the developed system were larger than the actual values of the leaf areas, especially when the leaf area was larger than 60 cm^2^. The relative error of the measured area reached the maximum value at the 72 cm^2^, while the other relative errors were less than 2%. These results indicated that the performance of the developed system may influenced by the environment.

### 3.5. Different Mobile Tests

**Figure 13:**
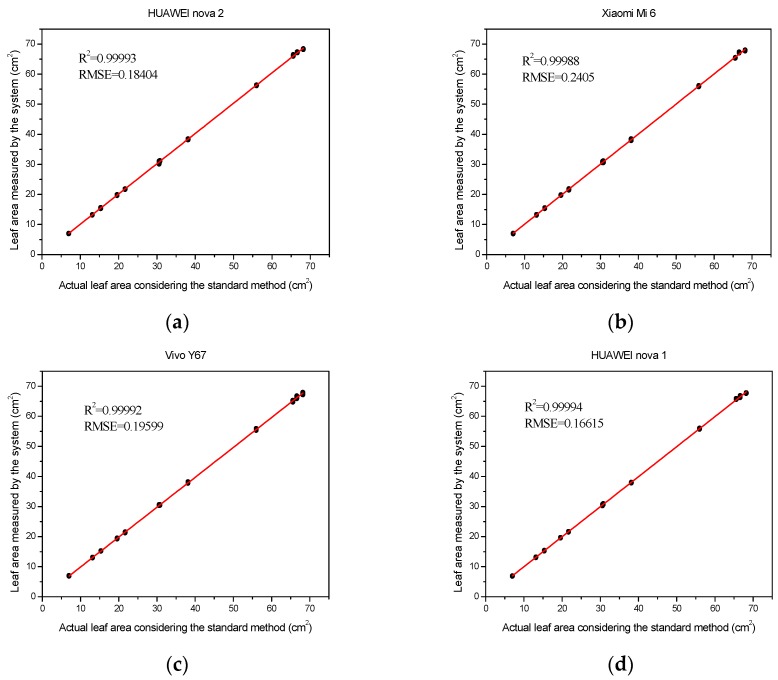
The relationship between leaf geometric parameters measured by the traditional method and by the developed system. (**a**) HUAWEI nova 2; (**b**) Xiaomi Mi6; (**c**) Vivo Y67; and (**d**) HUAWEI nova 1.

The resultant leaf areas, considering the values from the 12 leaves, were not significantly different between the mobiles, as demonstrated by the high *R*^2^ values (over 0.99) between the leaf areas from the phones and the standard method (Figure 13). The RMSE was considered high, regardless of the mobile used for measuring leaf area, the RMSE values were in the rage of 0.17–0.24. These results showed that all the four mobile phones performed well and had similar accuracy, in terms of measuring leaf areas.

## 4. Discussion

### 4.1. Effects of Test Distance on Geometric Parameters of Regular Images

The relative error was found to be the largest when the test distance was set at 230 mm and the error was reduced when the test distance was increased to 235 mm (Figure 7). This phenomenon could be related to the process of camera imaging. The closer the distance, the more the leaf edge was blurred, and the leaf edge was progressively getting closer to the background. During image processing, the actual pixel inside the leaf was removed, which resulted in calculation errors of total pixels of leaf area and other geometric parameters.

The measured results were influenced by graphic shape. The calculation of leaf area mostly adopted was the fixed reference method in previous studies [29], and the leaf area was determined by the proportion of pixels between the leaf and the reference object. However, in the actual measurement process, if the reference object was small, the actual size represented by a single pixel would weigh higher, and the reference object would have fewer pixels, and the conversion error of reference object would be larger; if the reference object was large, the actual size of a single pixel would be lower, and the conversion error would be also larger. Only the closer pixels of the reference object were to pixels of the measured leaf, the smaller the errors were. In order to solve the above problems, this study proposed that the positive circumscribed rectangle of the leaf as the reference object of the leaf. The reference object changed with the change in leaf size, and the reference object size and the leaf size were very close. The weight of the pixel in this image was also close, which avoided the measurement error caused by the reference object being too large or too small, which could significantly improve the measurement accuracy.

The method for measuring leaf parameters by the developed system was to use the positive circumscribed rectangle of measured object as a reference object. When the measured object was a rectangle, it was equivalent to using itself as a reference object, and the pixel of measured object was substantially the same as the pixel of the reference object. Therefore, the measurement results were also accurate.

### 4.2. Effects of Test Angle on Geometric Parameters of Regular Images

Different test angles had a certain influence on the graphic areas (Figure 8). As the test angle increased, image distortion worsened. The distorted image didn’t reflect the true condition of the graphic, and the measured area was not the true area of the graphic. As a result, if the relative errors of the measured geometric parameters increased, the relative errors would spread in a wider range. Since the pixel of distorted image was not necessarily an integer position in the corrected image, the corresponding pixel position might not be found, or missed. Although this study performed gray-scale interpolation on the vacant pixels, it would still lose some pixels, resulting in measured values being smaller than the true values.

Although the distortion calibration method was used in this study, it didn’t completely eliminate distortions. However, the relative errors were kept within 2% when the angle increased from 0 to 45 degrees, meaning that this distortion calibration method was feasible. Due to the fact that the correct distortion point could be found by the distortion calibration method in this paper. The Hough transformation method could extract the edge line of the rectangular reference object, and then find the intersection point of the line to obtain the corner point. In this method, the Hough transform parameters required by different images were different. If the Hough transform parameters were set incorrectly, some lines that did not belong to the rectangular frame might be found [30,31,32], or some edges might not be detected. In both cases, the rectangular frame was not extracted accurately, and the corner point couldn’t be obtained correctly [33].

### 4.3. The Relationship between the Measured Values of the Leaf geometric Parameters and the True Values from Lab Measurements

The coefficients of determination were as high as 0.99 (Figure 9a–d), meaning that the measurement accuracy of leaf parameters by this method was reasonably high. In this method, a positive circumscribed rectangle of the leaf was found to be the leaf reference. The number of pixels of the leaf reference was close to the number of pixels of the leaf, which avoided the calculation error caused by the difference between the reference pixel number and the leaf pixel number. The measured value of the leaf area was generally larger than the standard value (Figure 10), and this phenomenon could be partially explained by the edge shadow of the leaf. Plant leaves had a certain thickness [34]. The images of leaves obtained by the mobile phone camera often had shadows on the edges of leaves. The brightness of leaf edge shadow contrasted to the background largely, and the shadow was also classified into the leaf area when binarized, and the calculation for the pixels of the leaf area was larger than the actual number, causing that the measured values of leaves were larger than the actual leaf area. Moreover, because of leaves thickness increasing, the leaf edge shadow became larger and larger. Thus, the measured value was larger than the actual value especially when the leaf area is larger than 50 cm^2^.

The measured error of rice leaf area was the largest, because rice leaves were thin and easy to curl naturally. In the measurements of leaf area, the developed system was used first, followed by the graph paper method. After removing the leaf from rice, the water loss of leaf increased over time, causing leaf shrinking. As the results, the value measured by the graph paper method was lower than that measured by the developed system. Moreover, the rice leaves were slender and had an elongation axis, and it was difficult to be placed in parallel to the either sides of the correction plate, and the left and right borders of the obtained positive circumscribed rectangle of the leaf were also not parallel with the elongation axis of the rice leaf. Furthermore, the positive circumscribed rectangle of the rice leaf was large. All these contributed to the large relative errors of rice leaves.

### 4.4. The Relationship between the Measured Values of the Leaf Geometric Parameters and the True Values from Outdoor Measurements

The measured leaf parameters based on the positive circumscribed rectangle of the leaf and the measured leaf parameters using the standard method from outdoor measurements were closely correlated (*R*^2^ values higher than 0.99; Figure 11a–d). The relative errors of leaf area were less than 2.5%, meaning that the measurement accuracy was reasonably good when measuring outdoors. However, the illumination had some influences on the photographing scene in outdoor conditions, which might lead to occasional measurement errors. The natural light had a large effect on shadows. On cloudy days, light would scatter and diffuse, producing very soft shadow. Direct light, including direct sunlight, flash or tungsten light, would produce a hard, sharp-edged shadow that formed a strong contrast to the bright part of the object. In general, the stronger the sunlight was, the darker the shadow would be [34], the more likely the shadow would be seen as part of the leaf, and the greater the measurement error would be. Therefore, when the leaf area was 72 cm^2^, the error was the largest, it was likely because the light variance, leading to a lager shadow, and in turn a large error. Therefore, measurements should be performed under reasonable illumination to improve measurement accuracy and efficiency in outdoor conditions. Moreover, leaf edge shadows caused the measured value being larger than the actual value when the leaf area is larger than 60 cm^2^.

### 4.5. The Relationship between the Measured Values of Different Mobile Phones

Using different mobile phones to measure leaf area slightly affected the measurement results. Four different mobile phones with different resolutions were used to measure leaf areas. Optical resolution is a key attribute of the camera, which affects the quality of the images [35]. In general, images acquired with high-resolution devices have higher measurement accuracy. However, the sensors and lenses in different mobile phones are different, resulting in their inability to capture the same quality of the images even when the resolution was the same. Therefore, even with the highest resolution of HUAWEI nova 2, the highest measurement accuracy may not be obtained. Similarly, the HUAWEI nova 2 mobile has the lowest resolution and the measurement accuracy was not the worst.

### 4.6. Comparisons with Commercial Area Meters

The relative error of the leaf geometric parameters measurement with the commercial area meter ranged from 2% to 5%, whereas relative error of leaf geometric parameters in the present method was less than 2.5%, meaning that the leaf parameters measurement accuracy of the developed method was better. During measurements using the commercial area meter, the users had to keep a constant speed to pull down the leaf from the scanning plate. Keeping a constant speed was difficult in the field measurements, likely resulting in measurement errors. The present measurement leaf parameters based on positive circumscribed rectangle of the leaf did not need a constant speed. This allowed fully automatic leaf parameter calculations. Moreover, because the positive circumscribed rectangle of the leaf was used as the leaf reference, there were small differences in the number of pixels of the leaf. This led to more accurate leaf parameter calculations.

## 5. Conclusions

In this study, the Android mobile phone was used to process and measure geometric parameters of leaves, such as length, width, perimeter, and area. The image algorithms, distortion calibrations, and the method for calculating leaf parameters improved measurement accuracy. In addition, the parameter calculations based on the leaf contour did not require morphological processing of the leaf to eliminate holes and discontinuities. As a result, the leaf parameters measured by the method and standard method had a high correlation (*R*^2^ values were higher than 0.99), and the relative errors of leaf area were less than 2.5%. In addition, for the same leaf, the results of different mobile phone measurements were not significantly different. The main drawback exhibited by the proposed system is that the study did not consider the left and right borders of the obtained positive circumscribed rectangle of the leaf, and they were also not parallel with the elongation axis of the leaf, causing large relative errors. However, this system with acceptable accuracies achieved the objectives of this research study. The leaf geometry parameter measurement system based on the Android phone platform developed in this study was not only easy to operate, less affected by the distance, angle, leaf shape, and the environment, but also produced high measurement accuracy for leaf geometry parameters. In future studies, a method of leaf elongation axis extraction should be proposed to extract the circumscribed rectangle with left and right boundaries being parallel to the elongation axis of the leaf.

## Figures and Tables

**Table 1:** Specifications of Android phones.

Number	Phone model	CPU	Resolution	Android version
1	HUAWEI nova2s	Qualcomm snapdragon 855	4608 × 3456	8.0
2	Millet 6	Qualcomm snapdragon 845	4032 × 3016	8.1
3	Vivo Y67	Qualcomm snapdragon 855	4160 × 3120	6.0
4	HUAWEI nova1	Qualcomm snapdragon 625	3264 × 2248	7.0

**Table 2:** The specific shape and size of the regular leaf graphics.

Geometry	Length (cm)	Width (cm)	Area (cm^2^)	Perimeter (cm)
**Square**	5.00	5.00	25.00	20.00
**Rectangle**	6.00	4.00	24.00	20.00
**Circle**	6.00	6.00	28.27	18.85
**Triangle**	6.00	6.00	18.00	19.40
